# Adipose tissue dysfunction is associated with low levels of the novel Palmitic Acid Hydroxystearic Acids

**DOI:** 10.1038/s41598-018-34113-3

**Published:** 2018-10-25

**Authors:** Ann Hammarstedt, Ismail Syed, Archana Vijayakumar, Björn Eliasson, Silvia Gogg, Barbara B. Kahn, Ulf Smith

**Affiliations:** 1 The Lundberg Laboratory for Diabetes Research, Departments of Molecular and Clinical Medicine, Institute of Medicine, Sahlgrenska Academy at the University of Gothenburg, Gothenburg, Sweden; 2 Department of Medicine, Beth Israel Deaconess and Harvard Medical School, Boston, Massachusetts USA

## Abstract

Adipose tissue dysfunction is considered an important contributor to systemic insulin resistance and Type 2 diabetes (T2D). Recently, a novel family of endogenous lipids, palmitic acid hydroxy stearic acids (PAHSAs), was discovered. These have anti-diabetic and anti-inflammatory effects in mice and are reduced in serum and adipose tissue of insulin resistant humans. In the present study, we investigate if adipose tissue dysfunction is associated with reduced PAHSA levels in human subjects and if PAHSAs influence adipocyte differentiation. Our results show that low expression of adipocyte GLUT4 and adipocyte hypertrophy, markers of adipose tissue dysfunction, are associated with reduced expression of key enzymes for de novo lipogenesis and adipose tissue levels of PAHSAs in human subjects. We also show that GLUT4 is not only a marker of adipose tissue dysfunction, but may be causally related to the observed impairments. PAHSAs may also act locally in the adipose tissue to improve adipogenesis through a mechanism bypassing direct activation of peroxisome proliferator-activated receptor (PPARγ). The discovery of PAHSAs and our current results provide novel insights into positive effects of lipid species in adipose tissue and mechanisms by which dysfunctional adipose tissue is associated with insulin resistance and risk of developing T2D.

## Introduction

It is well documented that adipose tissue dysfunction, characterized by adipocyte hypertrophy and impaired adipogenesis, is an important contributor to systemic insulin resistance. Hypertrophic obesity has a central role in driving insulin resistance^1,2^ and has also been shown to be an independent predictor of future T2D development^3,4^. Conversely, adipose tissue of insulin-sensitive obese individuals is characterized by adipocyte hyperplasia and improved adipocyte differentiation compared to equally obese insulin-resistant individuals^5^.

In both human and mouse obesity GLUT4 is reduced in the adipose tissue but unchanged in skeletal muscle^6^. Mimicking this by deleting GLUT4 in mouse adipocytes results in whole body insulin resistance and increased diabetes risk^7^, whereas adipose tissue-selective overexpression of GLUT4 results in hyperplastic expansion of the adipose tissue and improved glucose metabolism^8,9^. GLUT4 is also reduced in the adipose tissue in human insulin-resistance and the degree of reduction correlates with whole-body insulin sensitivity^10^. These findings demonstrate that reduced GLUT4 levels are an important contributor to, and a marker of, adipose tissue dysfunction and its metabolic complications.

The beneficial metabolic effects seen in mice with adipose tissue-specific overexpression of GLUT4 (AG4OX) are related to increased glucose-dependent de novo lipogenesis in the adipose tissue^11^, and production of a novel class of lipids characterized by a branched ester linkage between a fatty acid and a hydroxy-fatty acid, FAHFAs^12^. In recent publications, we showed that the palmitic acid-hydroxy stearic acids (PAHSAs) have anti-diabetic and anti-inflammatory effects in mouse models of obesity and insulin resistance^13^. We also showed that PAHSA levels are reduced in the subcutaneous adipose tissue and in serum of insulin-resistant individuals suggesting that restoring PAHSA levels could have beneficial metabolic effects^12^. In agreement with this, insulin-resistant mice on a high fat diet were treated with PAHSAs and showed improvement in both insulin resistance and glucose tolerance^12,13^. Mechanistically, we showed that PAHSAs exert their effects, at least in part, through activation of the G-protein coupled receptor 120 (GPR120). This receptor has also previously been described to enhance adipocyte differentiation and improve metabolic health (reviewed in^14^). Interestingly, recent work has shown that the positive PAHSA effects on insulin secretion are mediated by GPR40^13^ and this receptor also appears to play a role in PAHSA effects on insulin sensitivity. Taken together, these and previous findings suggest a link between adipose tissue dysfunction and the low levels of PAHSAs observed in insulin-resistance. However, so far, very little is known about potential direct effects of PAHSAs in adipose tissue biology and human physiology.

The purpose of the present study is to investigate if adipose tissue dysfunction, characterized by adipocyte hypertrophy and markers of impaired differentiation, is associated with low level of PAHSAs in human subjects. In addition, we examined if PAHSAs have direct effects on adipocyte differentiation.

## Results

### Reduced GLUT4 is a marker of adipose tissue dysfunction and insulin resistance in man

**Figure 1:**
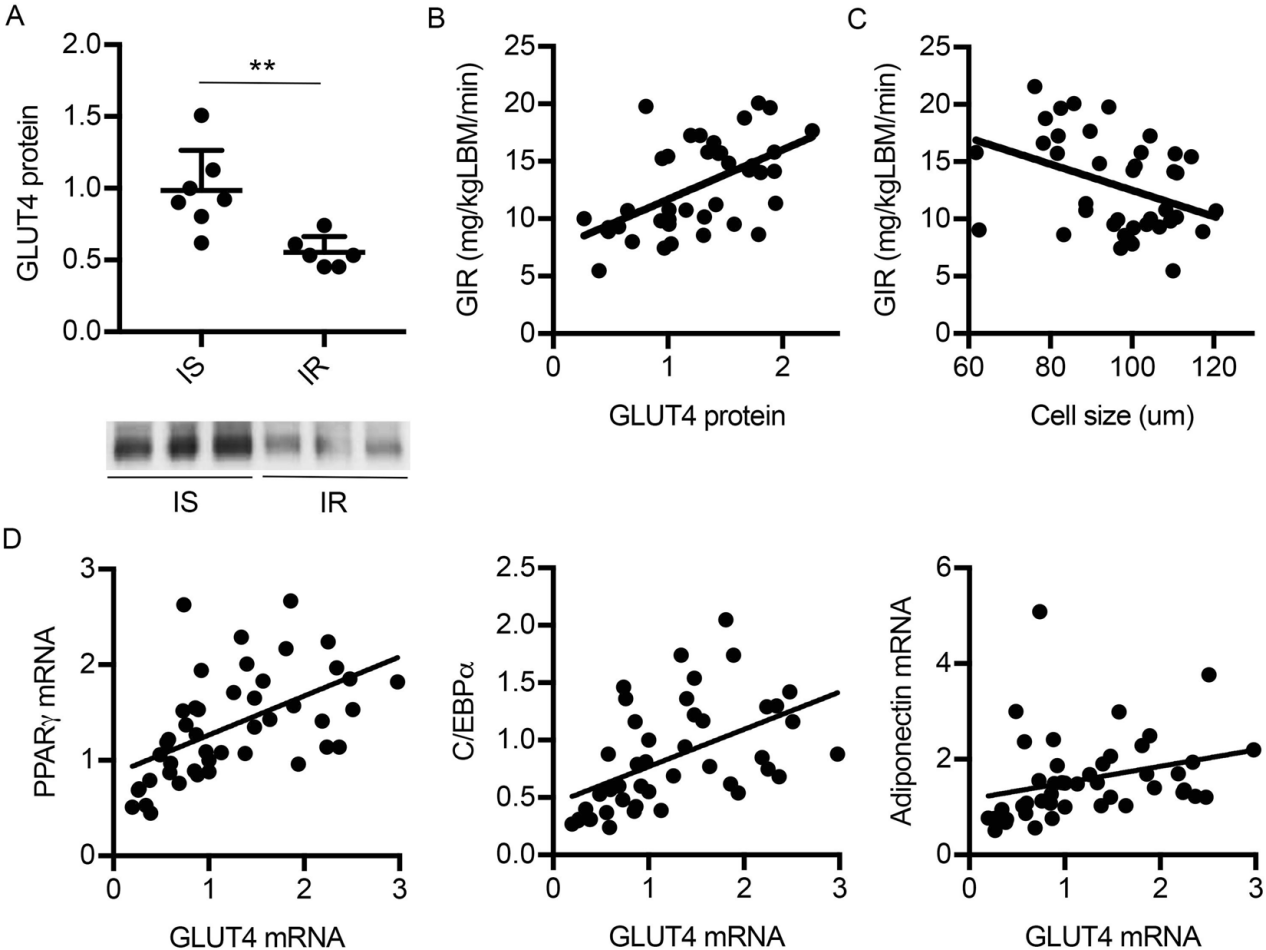
Adipose tissue dysfunction is associated with reduced insulin sensitivity – (**A**) Relative protein expression of GLUT4 determined by WB in isolated adipocytes from insulin sensitive (IS) and insulin resistant (IR) subjects. Data is presented as mean ± SEM (**B**) Relative protein expression of GLUT4 in relation to whole body insulin sensitivity measured by hyper-insulinemic euglycemic clamp. (**C**) Adipocyte cell size in relation to insulin sensitivity measured by hyper-insulinemic euglycemic clamp. (**D**) Relative expression of GLUT4 in relation to adipocyte differentiations markers PPARγ, C/EBPα and adiponectin. **p-value < 0.01.

We have previously shown that adipose tissue dysfunction is related to reduced whole body insulin sensitivity^10^. Both adipocyte hypertrophy and low expression of GLUT4 are markers of dysfunctional adipose tissue and inter-correlated^10^. However, it has not been investigated which of these two factors is the strongest predictor of whole-body insulin sensitivity. To answer this question, we performed a multiple regression analysis including adipocyte cell size and GLUT4 protein expression as predictors of insulin sensitivity measured by the hyperinsulinemic, euglucemic clamp technique in a cohort of non-diabetic subjects. As expected, GLUT4 protein was positively correlated with insulin sensitivity in two independent cohorts with similar range of insulin sensitivity (R = 0.56, p < 0.001) (Fig. 1A,B), while adipocyte cell size was negatively correlated with insulin sensitivity (R = −0.38, p = 0.017) (Fig. 1C). The standardized beta coefficient in the regression model indicated that GLUT4 protein expression is the stronger predictor of insulin sensitivity in the present cohort (Table 1).

**Table 1:** Multiple regression analysis – insulin sensitivity.

Independent variable	Dependent: Insulin sensitivity (GIR)				
	R	R^2^	Adj R^2^	F	p-value
**Model summary**					
GLUT4, Adipocyte size	0.60	0.36	0.33	10.02	<0.001
**Coefficients**					
	**B**	**SE**	**β**	**t**	**p-value**
Constant	12.93	4.54		2.85	0.007
GLUT4	4.09	1.09	0.53	3.75	0.001
Adipocyte size	−0.06	0.04	−0.19	−1.35	0.19

B, unstandardized coefficient; β, standardized coefficient Beta.

We further examined if GLUT4 expression also is a marker of adipose cell differentiation knowing that impaired adipocyte differentiation is an important part of the dysfunctional adipose tissue. We found GLUT4 mRNA levels to be positively correlated to other markers of adipogenic differentiation including peroxisome proliferator-activated receptor γ (PPARγ) (R = 0.629, p = < 0.001), CCAAT/enhancer binding protein (C/EBPα) (R = 0.269, p = < 0.001) as well as adiponectin (R = 0.483, p = 0.001) (Fig. 1E).

**Figure 2:**
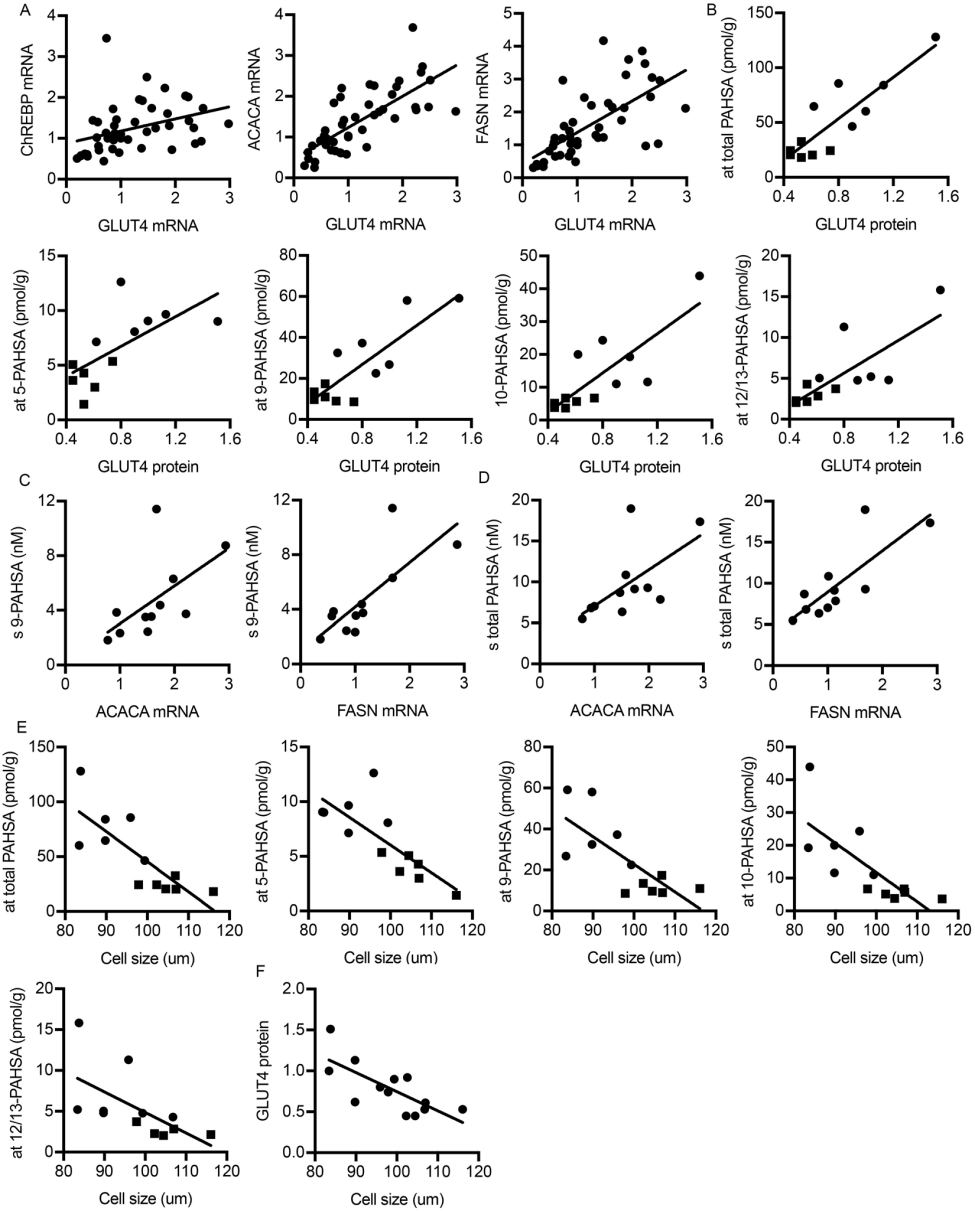
Adipose tissue dysfunction is associated with reduced lipogenesis and low adipose tissue PAHSA levels. (**A**) Relative quantification of GLUT4 mRNA measured in adipose tissue in relation to the lipogenic transcription factor ChREBP and its target genes ACACA and FASN. (**B**) Correlations of relative protein expression of GLUT4 determined by WB in isolated adipocytes in relation to different adipose tissue PAHSA isomers. Filled circles: subjects assigned to the IS group; filled squares: subjects assigned to the IR group. (**C**) Relative mRNA expression of the lipogenic enzymes ACACA and FASN in adipose tissue in relation to serum levels 9-PAHSA. (**D**) Relative mRNA expression of the lipogenic enzymes ACACA and FASN in adipose tissue in relation to serum levels of total-PAHSA (**E**) Correlations of adipocyte cell size and different adipose tissue PAHSA isomers. Filled circles: subjects assigned to the IS group; filled squares: subjects assigned to the IR group. (**F**) Correlation of GLUT4 protein expression and adipocyte cell size.

Previous studies in mice demonstrated that the positive metabolic effects of adipose cell GLUT4 overexpression were dependent on ChREBP; a key transcriptional regulator of *de novo* lipogenesis^15^. We therefore analyzed the expression of ChREBP and two central enzymes for *de novo* lipogenesis; acetyl-Co A carboxylase (ACACA) and fatty acid synthaste (FASN), in relation to GLUT4. As shown in Fig. 2A, all three lipogenic genes were positively and significantly correlated with GLUT4. In fact, ACACA and FASN were more strongly correlated to GLUT4 (R = 0.77, p =  < 0.001 and R = 0.734, p = < 0.001 respectively) than with ChREBP (R = 0.514, p = < 0.001) (Fig. 2A).

Both adipocyte differentiation markers and expression of lipogenic enzymes are related to adipocyte size. Thus, it is possible that there is no direct relationship to GLUT4, but rather a common factor related to adipocyte cell size. However, also after controlling for adipose cell size by means of partial correlation statistics the correlations between GLUT4 and adipocyte differentiation markers and lipogenic enzymes remained significant (Supplemental Table 1).

Thus, GLUT4 expression in the subcutaneous adipose tissue is a strong marker of several signs of a dysfunctional adipose tissue and, at least in this cohort, a stronger predictor of whole body insulin sensitivity than adipocyte size.

### GLUT4 protein correlates with PAHSA concentrations in human subcutaneous adipose tissue

We next asked if adipose tissue GLUT4 also is a marker of PAHSA isomer concentration in the adipose tissue and in the circulation in humans. Clinical and metabolic characteristics for this group are presented in our previous publication^12^. As shown in Fig. 2B, adipose tissue GLUT4 protein strongly correlates with all PAHSA isomers measured (5-PAHSA, R = 0.802, p = 0.002; 9-PAHSA, R = 0.687, p = 0.014; 10-PAHSA R = 0.564, p = 0.045; 12/13-PAHSA, R = 0.60, p = 0.03), as well as with total PAHSA levels (R = 0.739, p = 0.006) in the subcutaneous adipose tissue. These results suggest that GLUT4 is important for PAHSA production also in human adipose tissue.

In contrast, adipose tissue GLUT4 protein did not significantly correlate with serum levels of the different PAHSA isomers (data not shown) supporting that other cells/tissues, including the liver^12^, can also secrete PAHSAs and contribute to the circulating levels. However, significant correlations were seen for the lipogenic enzymes ACACA and FASN with circulating 9- (R = 0.709, p = 0.015 and R = 0.809, p = 0.003 respectively) (Fig. 2C) and total PAHSA levels (R = 0.69, p = 0.019 and R = 0.78, p = 0.004 respectively) (Fig. 2D).

Taken together, these data support that adipose tissue dysfunction is associated with reduced GLUT4, lipogenesis and PAHSA concentrations in insulin-resistant subjects.

### Adipocyte hypertrophy is associated with reduced adipose PAHSA levels

We next measured if adipocyte hypertrophy, as a marker of adipose tissue dysfunction, also is related to reduced PAHSA levels in the adipose tissue. As shown in Fig. 2E, there is a strong and inverse correlation between adipocyte cell size and all PAHSA isomers measured (5-PAHSA, R = −0.84, p < 0.001; 9-PAHSA, R = −0.72, p = 0.008; 10-PAHSA R = −0.83, p = 0.001; 12/13-PAHSA, R = −0.83, p = 0.03) including total levels (R = −0.837, p = 0.001). Also in this cohort, adipocyte hypertrophy was significantly associated with low GLUT4 protein levels confirming their close correlation (R = −0.73, p = 0.006) (Fig. 2F). After controlling for adipocyte size in this cohort, the correlations between GLUT4 protein and total-, 9- and 12/13-PAHSA were still significant (Supplemental Table 1). Aware of the limitations using this small cohort, we also performed a linear regression analysis to investigate whether GLUT4 protein expression or adipocyte size is the stronger predictor of total adipose tissue PAHSA levels in human adipocytes. The standardized beta coefficient indicates that GLUT4 protein expression is the stronger predictor (Table 2). Adipocyte cell size did not significantly correlate with serum levels of any of the PAHSAs measured (data not shown).

**Table 2:** Multiple regression analysis – adipose tissue PAHSA.

Independent variable	Dependent: Adipose tissue PAHSA				
	R	R^2^	Adj R^2^	F	p-value
**Model summary**					
GLUT4, Adipocyte size	0.89	0.80	0.75	17.73	0.001
**Coefficients**					
	**B**	**SE**	**β**	**t**	**p-value**
Constant	103.1	94.8		1.09	0.305
GLUT4	68.94	25.37	0.63	2.72	0.024
Adipocyte size	−1.08	0.80	−0.31	−1.34	0.21

B, unstandardized coefficient; β, standardized coefficient Beta.

### Silencing GLUT4 impairs adipocyte differentiation

The fact that adipose tissue-specific overexpression of GLUT4 drives hyperplastic expansion of the adipose tissue^8,9^ together with its strong positive correlation with adipocyte differentiations markers, lipogenesis and adipose tissue PAHSA concentrations made us hypothesize that GLUT4 is not merely a marker of dysregulated adipose tissue with impaired adipocyte differentiation, but may be a central to adipose cell differentiation.

**Figure 3:**
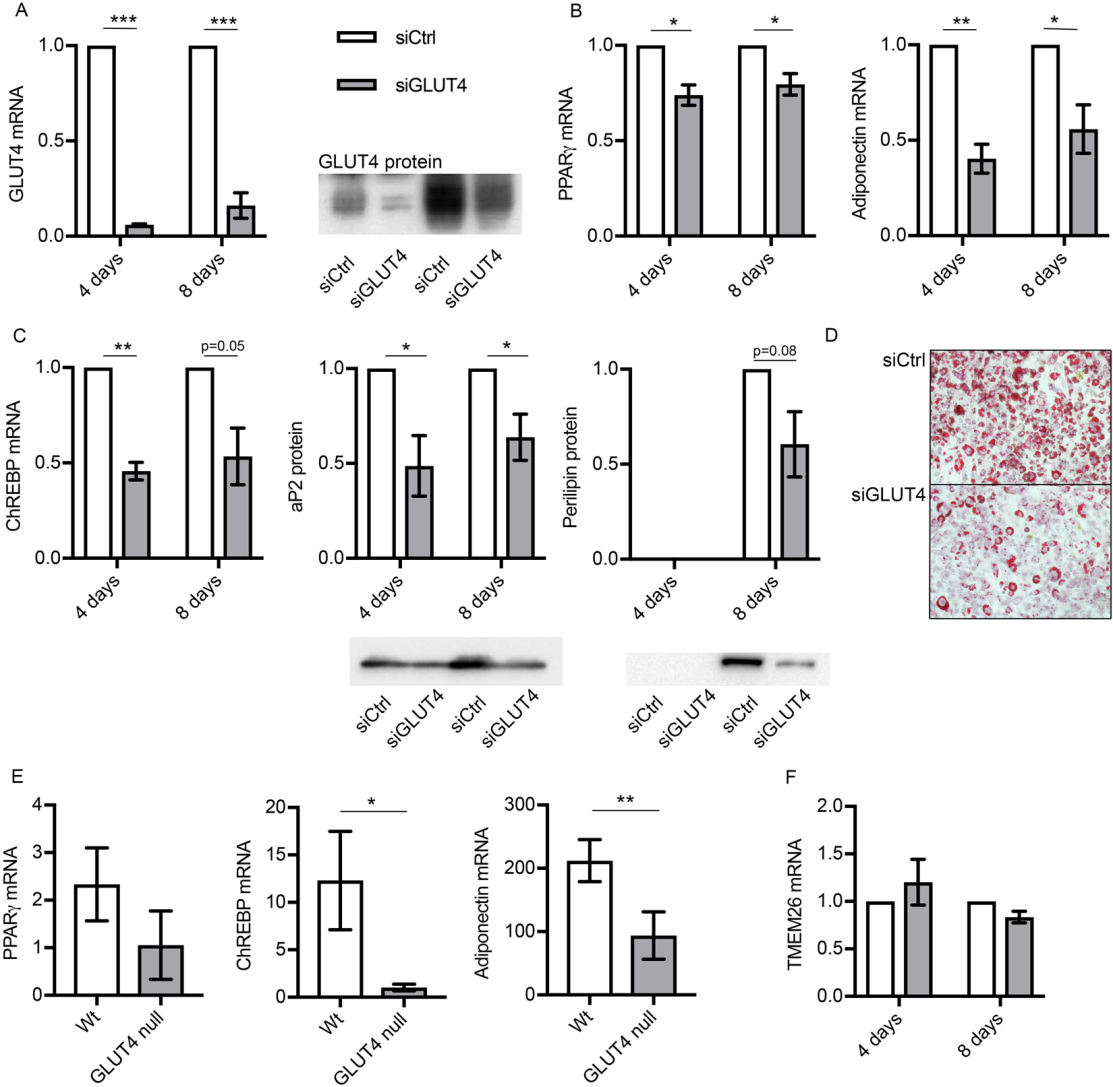
Silencing GLUT4 results in impaired adipocyte differentiation. (**A**) Relative gene expression of GLUT4 in 3T3-L1 pre-adipocytes in the presence of anti-GLUT4 siRNA or scrambled at day 4 and 8 of differentiation. (**B**) Relative gene expression of adipocyte differentiation markers PPARγ and adiponection in the presence of anti-GLUT4 siRNA or scrambled at day 4 and 8 of differentiation. (**C**) Relative gene and protein expression of ChREBP, aP2 and perilipin in the presence of anti-GLUT4 siRNA or scrambled at day 4 and 8 of adipocyte differentiation. (**D**) Lipid accumulation visualized by Oil Red O staining in the presence of anti-GLUT4 siRNA or scrambled at day 8 of adipocyte differentiation. (**E**) Relative gene expression of PPARγ, ChREBP and adiponectin and Oil Red O staining of *in vitro* differentiated adipocytes from wt and GLUT4ko mice. (**F**) Relative gene expression of TEMEM26 in the presence of anti-GLUT4 siRNA or scrambled at day 4 and 8 of differentiation. Data is presented as mean ± SEM related to control siRNA, n = 4–6. *p-value < 0.05, **p-value < 0.01, ***p-value < 0.001.

To answer this question, we silenced GLUT4 in 3T3-L1 pre-adipocytes undergoing differentiation. As shown in Fig. 3A, silencing with specific siRNAs resulted in a 94% reduction of GLUT4 after 4 days of differentiation. Although the endogenous gene and protein expression of GLUT4 increased during progression of adipocyte differentiation, anti-GLUT4 siRNA remained effective even eight days after differentiation reducing the levels of GLUT4 to approximately 16% of control levels.

Silencing GLUT4 results in impaired adipocyte differentiation as evident by reduced expression of PPARγ and the differentiation marker adiponectin (Fig. 3B). Furthermore, genes important for lipogenesis and carbohydrate metabolism (ChREBP) and lipid accumulation (aP2 and perilipin), were also reduced (Fig. 3C). In line with these findings, lipid accumulation at day eight was also reduced as shown by Oil Red O staining (Fig. 3D). Unfortunately, we were not able to examine if silencing GLUT4 resulted in reduced concentration of PAHSA since the concentrations in 3T3L1 cells in are below a reliable detection level. The data regarding reduction of Glut4 expression in 3T3-L1 cells were further supported by analyzing primary mouse pre-adipocytes from GLUT4 knock-out mice. Differentiation of preadipocytes lacking GLUT4 into adipocytes resulted in reduced expression of both ChREBP and adiponectin compared to control cells expressing GLUT4. PPARγ mRNA expression also tended to be lower in adipocytes lacking GLUT4 although this did not reach statistical significance (Fig. 3E).

A possible explanation of the impaired differentiation and lipid accumulation in the absence of GLUT4 could be lack of glucose/fuel entering the cell. However, there was no sign of a compensatory increase in GLUT1 expression (data not shown), which previously has been shown to increase in response to starvation of 3T3-L1 cells^16^.

Furthermore, to address the possibility that the reduced lipid accumulation was due to increased oxidative capacity/browning of the adipocytes, we measured gene expression of uncoupling protein-1 (UCP-1), PRD1-BF1-RIZ1 homologous domain containing 16 (PRDM16) and transmembrane protein 26 (TMEM26). While, UCP-1 and PRDM16 were barely detectable at any condition, TMEM26 was not changed by GLUT4 silencing (data not shown and Fig. 3F).

### PAHSAs promote adipocyte differentiation

We then asked if PAHSAs, secreted by the adipose tissue, can have auto-/paracrine effects on adipocyte differentiation. To answer this, we differentiated 3T3-L1 mouse pre-adipocytes and human primary pre-adipocytes to mature adipocytes in the presence or absence of added 5- and 9-PAHSAs.

**Figure 4:**
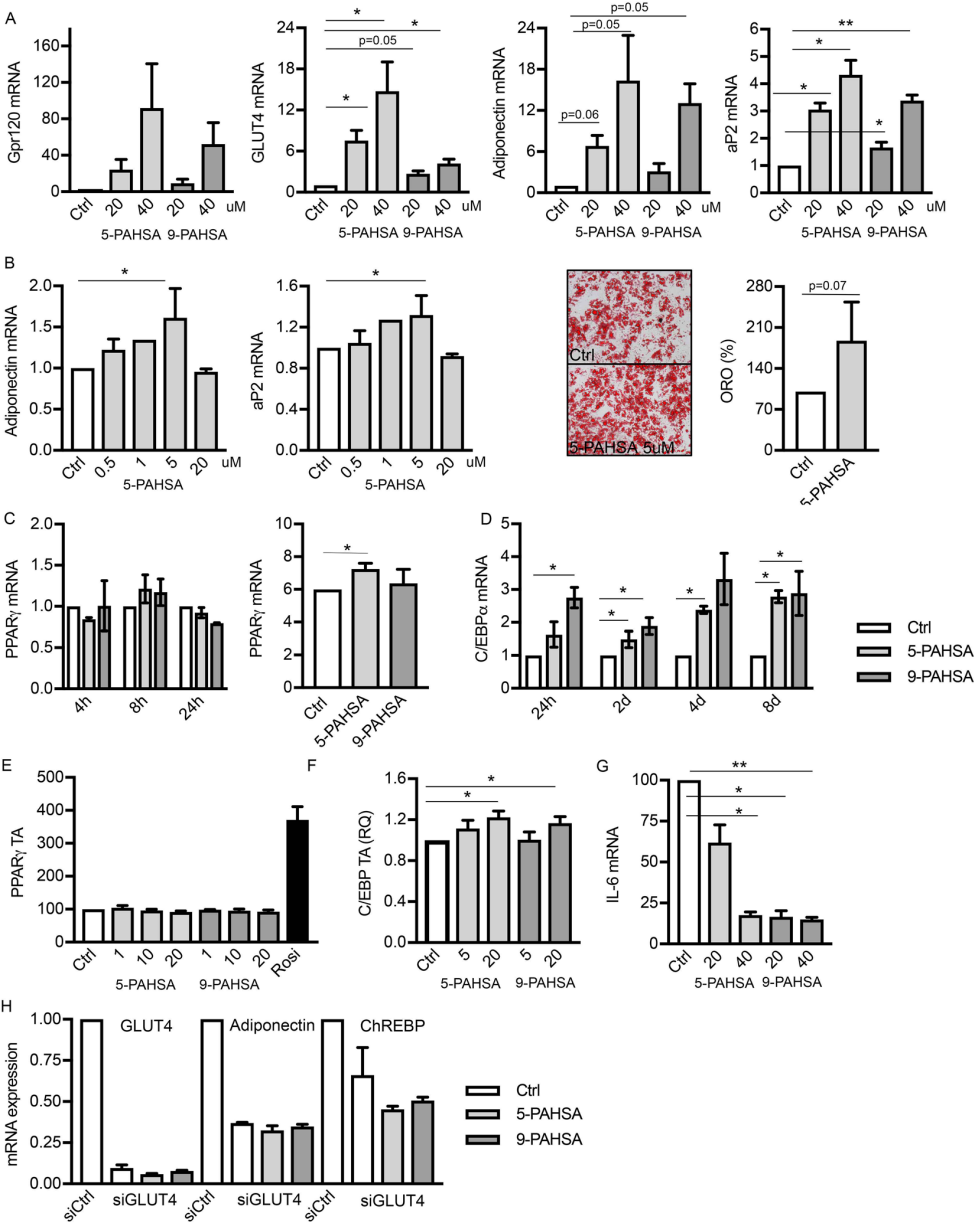
Addition of 5- and 9-PAHSA to pre-adipocytes promote adipocyte differentiation. (**A**) Relative gene expression of gpr120, GLUT4, adiponectin and aP2 in the presence or absence of 5- and 9-PAHSA during adipocyte differentiation. (**B**) Relative gene expression of adiponectin and aP2 and Oil Red O staining in the presence or absence of 5-PAHSA during differentiation of primary human pre-adipocytes. (**C**) Relative gene expression of PPARγ in the presence or absence of 5- and 9-PAHSA during the first 24hrs of adipocyte differentiation and AUC for time points 0, 2, 4 and 8 days. (**D**) Relative gene expression of C/EBPα in the presence or absence of 5- and 9-PAHSA at 24hrs and 2, 4 and 8 days. (**E**) Transcriptional activity of PPARγ in the presence of 1, 10 and 20uM 5- or 9-PAHSA. (**F**) Transcriptional activity of C/EBPs in the presence of 5 and 20uM 5- or 9-PAHSA. (**G**) Relative gene expression of IL6 at day 2 of adipocyte differentiation in the presence or absence of 5- and 9-PAHSA (**H**) Relative gene expression of GLUT4, Adiponectin and ChREBP in the presence of scrambled or anti-GLUT4 siRNA ± 5-and 9-PAHSA (20uM). Data is presented as mean ± SEM related to control, n = 3–6. *p-value < 0.05, **p-value < 0.01.

As shown in Fig. 4A, both 5- and 9-PAHSA enhanced adipogenesis in 3T3-L1 pre-adipocytes and dose-dependently increased the expression of several differentiation and insulin sensitivity markers such as GPR120, GLUT4, adiponectin and aP2. In addition, genes involved in lipid transport/metabolism CD36, FABP4 and FASN were significantly upregulated in the presence of PAHSAs (data not shown). Similar data was obtained from primary human pre-adipocytes treated with 5-PAHSA during adipocyte differentiation although there were larger inter-individual differences as usually seen with human cells (Fig. 4B).

### The PAHSA-promoting effect on adipogenesis is not related to PPARγ transcriptional activation

To address potential mechanisms for the enhanced adipogenesis in the presence of PAHSAs, we examined the expression profile of key early adipogenic transcription factors. Surprisingly, the effect on PPARγ expression was limited (Fig. 4C), while C/EBPα was significantly increased at all time points examined (Fig. 4D). C/EBPα was not expressed at the early time points 4 or 8 hours after induction of differentiation.

Since the transcriptional activation of PPARγ is not necessarily directly related to function, we investigated the possibility that PAHSAs act as endogenous PPARγ ligands increasing its transcriptional activity. To address this, we used HEK-293 cells containing a PPARγ-GAL4 DNA binding fusion protein reporter system and monitored the beta-lactamase enzymatic activity in the presence of different concentrations of PAHSAs.

Addition of rosiglitazone, a known PPARγ ligand, results in robust activation of PPARγ. However, neither 5-PAHSA nor 9-PAHSA enhanced transcriptional activation of PPARγ at any of the concentrations used (Fig. 4E). Thus, these data show that PAHSAs enhance adipogenesis but not through direct PPARγ activation.

Since transcriptional activity of C/EBPα has been shown to be essential for full adipocyte differentiation and function, including acquisition of insulin sensitivity^17^, we also examined if the PAHSAs could activate C/EBPs. We used HEK293 cells transfected with a Luciferase reporter system containing C/EBP binding sites and monitored its activity in the presence of 5- and 9-PAHSA. Our results show that the transcriptional activation of C/EBPs was enhanced in the presence of PAHSAs (Fig. 4F).

In addition to the pro-adipogenic effects of 5- and 9-PAHSA, we also saw reduced expression of IL-6 during the early stages of adipocyte differentiation in 3T3-L1 cells. (Fig. 4G). IL-6 is a cytokine known to inhibit adipocyte differentiation^18^, suggesting that PAHSAs may also promote adipocyte differentiation through downregulation of this cytokine.

Together, these data suggest that PAHSAs produced by the adipose tissue, through paracrine effects, can promote differentiation of pre-adipocytes and enhance the ability of adipose tissue to store lipids. These findings open up a new possible mechanism for how PAHSAs improve whole-body insulin sensitivity.

### PAHSAs do not prevent the negative effect of GLUT4 silencing on adipocyte differentiation

In light of the positive effects of PAHSAs on adipocyte differentiation, we investigated if addition of PAHSAs could rescue the impaired adipogenesis following GLUT4 silencing.

We silenced GLUT4 before and during adipocyte differentiation of 3T3-L1 pre-adipocytes with and without addition of 5- and 9-PAHSA to the culture media. As shown in Fig. 4H, addition of 5- and 9-PAHSA did not overcome the negative effects of GLUT4 silencing on adipogenesis including transcriptional activation of adiponectin and ChREBP. These findings support the presence of additional factors regulated by GLUT4 that affect adipose cell differentiation which could include other FAHFA isomers. This needs to be directly studied.

## Discussion

We hypothesized in the present study that markers of human subcutaneous adipose tissue dysfunction, including reduced GLUT4, adiponectin and lipogenic enzymes, were associated with low tissue levels of PAHSAs and our current results document this. We found strong correlations between these markers of adipose tissue dysfunction and low levels of all adipose tissue PAHSA isomers measured. Adipose tissue expression of key de novo lipogenic enzymes was highly correlated with circulating levels of 9- and total-PAHSA. This suggests an important role of adipose tissue lipogenesis in regulating circulating PAHSA levels which is consistent with our previous report of reduced PAHSA levels in adipose tissue and serum of adipose-ChREBP knockout mice^19^. The adipose tissue PAHSA concentrations are expressed per mg tissue. It is possible that the results would be different if the concentrations were expressed per mg of protein. However, previous publications show that protein content is either unchanged or positively correlated with adipocyte size^20–23^, which would strengthen the correlations we observe. In addition, since PAHSAs are products of an enzymatic pathway, and is likely that their abundance correlates with the activity of the specific pathway rather than with total protein content. Thus, most likely adjusting for total protein content would not change the overall results. However, this question can be addressed in future studies.

The lack of significant correlations between GLUT4 and adipocyte size with circulating PAHSA levels may be explained by the small study cohort since trends could be seen for some serum PAHSAs. Most importantly, however, adipose tissue is not the only tissue producing PAHSAs^12^ and the serum contribution of adipose-derived PAHSAs may be diluted by other secreting tissues.

In our current study, we also show that experimentally reducing the cellular level of GLUT4, similar to that seen in insulin-resistance in both man and murine models, results in pronounced impairment of adipocyte differentiation that did not result from nutrient deprivation. These findings suggest that low adipose tissue GLUT4 is not merely a marker of a dysfunctional adipose tissue, but may in fact be a central contributor to the impaired adipogenesis and low adipose tissue PAHSA concentrations seen in hypertrophic adipose tissue. In fact, in the current cohort the expression of GLUT4 was a stronger predictor of insulin sensitivity than adipocyte size. This finding is in line with the observations that adipose tissue specific overexpression of GLUT4 in mice leads to improved glucose homeostasis in spite of increased fat mass. It also confers protection against high fat diet-induced impaired glucose tolerance which may result from increased PAHSAs with improved pre-adipocyte differentiation and hyperplastic adipose tissue expansion^8,11^.

The mechanisms for the reduced adipose tissue GLUT4 in insulin resistance can only be speculated upon. There may be contributing genetic factors since GLUT4 is reduced in adipose tissue in first-degree relatives to patients with T2D (FDRs) long before diabetes develops^24^. However, GLUT4 itself is not one of the identified diabetes risk genes. Furthermore, the fact that FDRs have a greater risk of developing diabetes than the pooled risk of all these genes^25^, suggests that the reduction can be due to the presence of increased inflammation and/or epigenetic regulation. Indeed, epigenetic regulation of GLUT4 by miRNA93 has been demonstrated in adipocytes in other conditions of insulin resistance and adipocyte hypertrophy^26^.

Large efforts have been made to understand the mechanisms favoring hyperplastic or hypertrophic adipose tissue expansion. We have shown that, for a given BMI, there are large inter-individual differences in subcutaneous adipocyte cells size and that this is related to the ability of the pre-adipocytes to undergo adipocyte differentiation, i.e., an individual whose pre-adipocytes differentiate poorly primarily expands his/her adipose tissue by cell hypertrophy, while individuals whose pre-adipocytes differentiate well may expand his/her adipose tissue by hyperplasia^27^. These findings are in line with observations by Arner *et al*. showing reduced turnover of adipose cells in hypertrophic adipose tissue^1^.

The results of our present study show that the novel PAHSA lipids, which are produced by the adipose tissue, can regulate the ability of pre-adipocytes to differentiate into mature adipocytes at least partly by activation of the C/EBP pathway. These results suggest that individuals with low adipose cell GLUT4 and endogenous PAHSA levels in the adipose tissue, also have reduced ability to differentiate new adipocytes, thus contributing to adipose tissue dysfunction as part of a negative circuit.

Although PAHSAs were shown to promote adipogenesis, the addition of the tested lipids could not rescue the impaired adipocyte differentiation induced by GLUT4 silencing. Modulating the level of adipose tissue GLUT4 profoundly affects fatty acid synthesis^11,12^. Thus, it is possible that silencing GLUT4 during adipocyte differentiation impairs the production of several PAHSAs or FAHFAs other than those tested that promote adipocyte differentiation through activation of PPARγ^28^. The beneficial effect of the PAHSAs tested on adipocyte differentiation is clearly not a direct effect on the transcriptional activity of PPARγ.

In conclusion, adipose tissue dysfunction, defined by adipocyte hypertrophy, low expression of cellular GLUT4, adiponectin and lipogenic enzymes, also is associated with low levels of adipose tissue PAHSA. Reduced GLUT4 may indeed be an important primary regulator of these downstream effects also involving currently unknown specific lipogenic enzymes for the formation of PAHSAs. Although further investigations are necessary to understand the underlying mechanisms, the discovery of PAHSAs and our current results provide novel insights into the beneficial effects of these lipids and why dysfunctional adipose tissue is associated with insulin resistance and risk of developing T2D.

## Methods

### Subjects

87 subjects with varying degrees of insulin sensitivity and BMI were recruited through advertisement in local media. Height and weight were measured and BMI calculated as kg body weight divided by height (m) squared. To evaluate insulin sensitivity a hyperinsulinaemic euglycaemic clamp was performed with an insulin infusion rate of 40 mU/m^2^ per min, as previously described^29^. Clinical and metabolic characteristics for this group are given in Table 3. Due to limited tissue availability, tissue from 41 subjects were used for adipocyte size measurements and GLUT4 protein quantification while tissue from 46 subjects was used for mRNA extraction.

**Table 3:** Clinical and metabolic characteristics of study population.

Variable	Mean ± SD
Gender (F/M)	44/43
Age (year)	41 ± 6
BMI (kg/m^2^)	25.9 ± 3.6
GIR (mg/kgLBM/min)	12.3 ± 4.0
Adipocyte diameter (um)	95 ± 13.5

BMI, body mass index; GIR, glucose infusion rate during hyperinsulinemic euglucemic clamp; LBM, lean body mass.

Thirteen additional subjects were part of our previous publication identifying FAHFAs as novel and metabolically important endogenous lipids. PAHSA levels in relation to insulin sensitivity were reported therein^12^.

Informed consent was obtained from all subjects prior to study start. All subjects received oral and written information and gave oral and written consent to participate.

### Lipid extraction and targeted LC/MS analysis

Lipid extraction and analysis were performed as described^12^. Briefly, tissues were Dounce homogenized on ice in a mixture of 1.5 ml MeOH, 1.5 ml chloroform and 3 ml citric acid buffer. PAHSA standards were added to choloroform prior to extraction. The resulting mixture was centrifuged and the organic phase containing extracted lipids was dried under N_2_ and stored at −80 °C prior to solid phase extraction.

PAHSAs were measured on an Agilent 6410 Triple Quad LC/MS via Multiple Reaction Monitoring in negative ionization mode: Extracted and fractionated samples were reconstituted in 26uM MeOH; 10ul was injected for analysis. A Luna C18(2) column was used with an in-line filter (Phenomenex). Distinct PAHSA species were resolved via isocratic flow at 0.2 ml/min for 120 min using 93:7 MeOH:H_2_0 with 5 mM ammonium acetate and 0.01% ammonium hydroxide as solvent.

### Adipose tissue biopsy

Subcutaneous adipose tissue biopsies were obtained from the periumbilical, abdominal region after an overnight fast. Tissue was snap frozen for subsequent RNA isolation or used for adipocyte isolation as described previously^30^. Briefly, biopsies were washed to remove traces of blood and treated with 0.8 mg/ml collagenase (Sigma, St Louis, MO, USA) for ∼60 min at 37 °C. Isolated adipose cells were filtered through a 250 μm nylon mesh, washed four times with fresh medium for removal of collagenase. Cell size was measured and remaining isolated adipocytes were used for protein extraction^31^.

### Cell culture and pre-adipocyte differentiation

For adipocyte differentiation 3T3-L1 fibroblasts were grown to confluence. Two days post-confluence differentiation was initiated by culturing cells in DMEM containing 10% fetal bovine serum (FBS), 0.5 mM methyl-isobutyl-xanthine, 1 μM dexamethasone and 865 nM insulin for 48 hrs, followed by insulin alone for an additional 48 hrs after which medium was removed and replaced with DMEM supplemented with 10% FBS until full differentiation. Cells were treated with compound or vehicle (DMSO) control during the entire differentiation protocol.

Differentiation of human preadipocytes was induced with a differentiation cocktail consisting of 850 nM insulin, 10 μM dexametasone, 0.5 mM isobutylmethylxanthine (IBMX), 10 μM pioglitazone in DMEM/F12 supplemented with 3% FBS. After three days, the medium was changed to a medium containing only insulin, dexamethasone and pioglitazone in DMEM/F12, 10% FBS. Cells were treated with compound or vehicle (DMSO) control during the last three days of the differentiation protocol.

Stromavascular-derived preadipocytes were isolated and differentiated from WT and Glut4 null mice as previously published^19^.

### Generation of Glut4 null mice

Glut4 Null mice were generated by crossing Glut4 lox male mice^32^ to ZP3- Cre female mice (zona pellucida 3- Cre purchased from Jackson Laboratory). Expression of Cre recombinase occurs in fertilized oocyte prior to the completion of the first meiotic division. By crossing Glut4 lox males to ZP3cre female, we obtained whole body Glut4 heterozygous (Glut4 Het) mice. The ZP3-cre was outbred and Glut4 Null mice were obtained by crossing Glut4 Het to each other. Mice have a mixed genetic background FVB x C57Blk/J. Littermate wild type mice were used as controls.

### GLUT4 silencing

For silencing of GLUT4 3T3-L1 pre-adipocytes were transfected with a heterogeneous mixture of anti-GLUT4 siRNA (EMU086551, Sigma-Aldrich) or control siRNA (EHUFLUC, Sigma-Aldrich) using Lipofectamine RNAiMax (Thermo Fischer Scientific) 96 hrs before initiation of differentiation.

### Oil Red O staining

To visualize lipid accumulation, differentiated cells were fixed with 4% formalin for 30 min, incubated with 60% isopropanol and stained with Oil Red O for 5 min. The stained area was quantified in three randomly selected field per experiments and quantified by using ImageJ software (ImageJ, NIH, USA).

### Cell extracts and Western blots

Cells were lysed in lysis buffer as described^18^, insoluble material sedimented by centrifugation, supernatants were collected and stored at −80 °C. Protein content was determined using a BCA Protein Assay kit (Pierce, Rockford, IL, USA). 20 μg protein from each sample was separated on 4–12% NuPAGE (Invitrogen, ThermoFisherScientific, Wilmington,USA) and transferred to nitrocellulose membranes (Schleicher & Schuell, BioSCience, Gmbh, Germany). Membranes were blocked for 1 h in 5% dry milk in PBS-Tween and incubated with specific primary antibodies; GLUT4 polyclonal antiserum kindly provided by Dr Sam Cushman, NIH; aP2 #3544; perilipin #9346 (both Cell Signaling Technology, Beverly, MA, USA) overnight followed by incubation with secondary antibodies according to the manufacturer’s instructions. Bands were visualized with ECL reagents (Amercham Biosciences Ltd., UK). Quantification of GLUT4 protein was standardized by loading a reference sample on each gel. A relative value obtained by dividing each sample with the reference sample was obtained and used for all analyses. Different reference samples have been used for the two cohorts and GLUT4 levels can therefore not be directly compared between cohorts.

### Quantitative real-time PCR

RNA was extracted using Qiagen RNeasy (Lipid Tissue) kit (Qiagen Gmbh, Hilden, Germany). Gene expression was analyzed with the Quant Studio 6 Flex sequence detection system (Applied biosystems, Foster City, CA, USA). Gene-specific primers and probes were designed using the Primer Express software (Applied biosystems, Foster City, CA, USA) and are available upon request. Each sample was run in duplicates and the quantity of a particular gene in each sample was normalized to ribosomal 18 s RNA.

### PPARγ reporter assay

To assess the transcriptional activity of PPARγ GeneBLAzer UAS-bla HEK 293 H cells stably expressing a beta-lactamase reporter gene was used according to the manufacturer’s instructions (Life Technologies, Europe).

Briefly, PPARγ-UAS-bla HEK 293 H cells were plated in a 384 well plate. The assay media w/o additions were added to the cells and incubated for 16 h. Rosiglitazone and the specific PPARγ inhibitor GW9662 were used as positive and negative control respectively.

The plate was read in a fluorescence plate reader (Infinite F200 Tecan, Austria) using 409-nm excitation and 460-nm (blue) and 530-nm (green) emissions through the clear bottom of the plate. The data were plotted as a “blue/green ratio” (460 nm/530 nm) after background subtraction.

### C/EBP reporter assay

To assess the transcriptional activity of C/EBPalpha we used the Dual-Luciferase Reporter Assay System in HEK 293 cells. Briefly, cells were transiently transfected with a luciferase reporter plasmid containing C/EBP response elements and a constitutively active renilla reporter plasmid as internal control of transfection efficiency (both Promega, Madison, WI, USA). 24hrs after transfection media was changed and additions made. After additional 24hrs Luciferase and Renilla activity was measured using a luminometer (Infinite F200 Tecan, Austria). The transcriptional activity of C/EBP was expressed as the ratio between Luciferase and Renilla for all conditions.

### Statistics

Data is presented as mean ± SEM or SD as indicated. Statistical significance between groups was evaluated using ANOVA, nonparametric Wilcoxon signed-rank test and TTEST as appropriate. Correlation between two parameters was assessed by Spearman´s rank correlation analysis and partial correlation. For estimation of the stronger predictive variable multiple regression was used to obtain the standardized Beta coefficient. A two tailed P-value < 0.05 was considered statistically significant. Statistical analyses were performed using SPSS statistics (v22, IBM corp, Armonk, NY, USA).

### Study approval

The human study protocol was approved by the local Ethical Committees at the Sahlgrenska Academy at the University of Gothenburg and was performed in accordance with the Declaration of Helsinki.

All animal care and use procedures were in accordance with and approved by the Institutional Animal Care and Use Committee at Beth Israel Deaconess Medical Center, Boston, MA.

## Electronic supplementary material

Supplementary Information
