# Supplementary material for: Adipose tissue dysfunction is associated with low levels of the novel Palmitic Acid Hydroxystearic Acids
Source: Sci Rep. 2018 Oct 25;8:15757. doi: 10.1038/s41598-018-34113-3 (PMC6202399; doi:10.1038/s41598-018-34113-3)
Supplement: Supplementary file 1 — Supplementary Information [file 41598_2018_34113_MOESM1_ESM.pdf]

Adipose tissue dysfunction is associated with low levels of the novel Palmitic Acid Hydroxystearic Acids

Ann Hammarstedt[1*], Ismail Syed[2], Archana Vijayakumar[2], Björn Eliasson[1], Silvia Gogg[1], Barbara B. Kahn[2] and Ulf Smith[1]

[1]The Lundberg Laboratory for Diabetes Research, Departments of Molecular and Clinical Medicine, Institute of Medicine, Sahlgrenska Academy at the University of Gothenburg, Gothenburg, Sweden. [2]Department of Medicine, Beth Israel Deaconess and Harvard Medical School, Boston, Massachusetts, USA.

[*]Corresponding author:

Ann Hammarstedt, PhD

The Lundberg Laboratory for Diabetes Research

Blå Stråket 5, Sahlgrenska University Hospital

413 45 Göteborg

Sweden

E-mail: Ann.hammarstedt@medic.gu.se

Phone: +46-31 342 6646

Supplemental table 1. Partial correlations controlling for adipocyte cell size

Gene vs GLUT4	R_p	*p-value*
PPARγ	0.50	0.001
C/EBPα	0.48	0.001
Adiponectin	0.75	<0.001
ChREBP	0.32	0.034
ACACA	0.75	<0.001
FASN	0.65	<0.001
PAHSA vs GLUT4	R_p	*p-value*
Total-PAHSA	0.67	0.024
5-PAHSA	0.18	0.61
9-PAHSA	0.63	0.039
10-PAHSA	0.54	0.087
12/13-PAHSA	0.61	0.049

Full length captures

<u>Full length membarnes to Figure 3A</u>
Some of the original membranes have been cut to enable blotting of multiple proteins at different molecular size.
The original tiff-files from caputre is full length.

Figure 3A GLUT4 protein

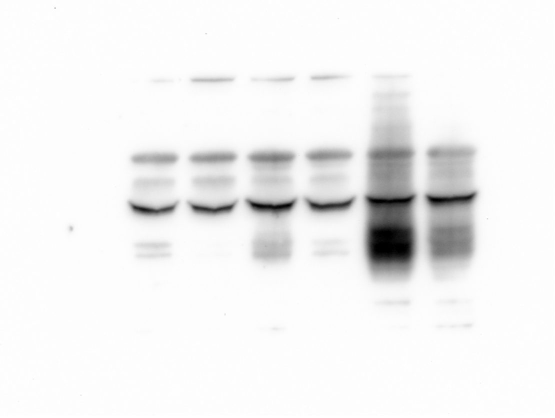

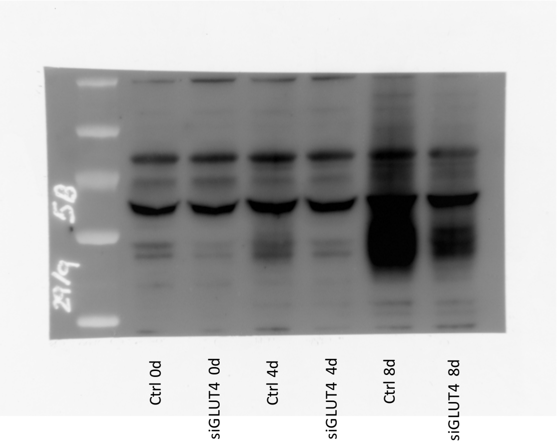

<u>Full length membarnes to Figure 3C</u>
FABP4 protein

Some of the original membranes have been cut to enable blotting of multiple proteins at different molecular size.
The original tiff-files from caputre is full size.

Two independent experiments have been visulized at the same time -> both are on the original TIFF-file

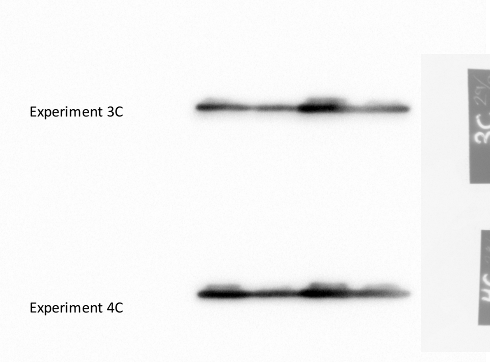

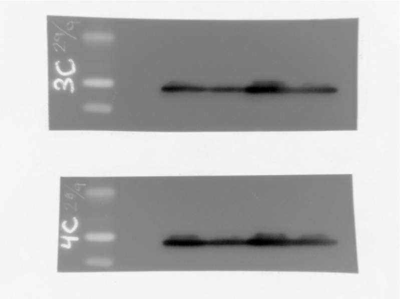

All quantified blotts

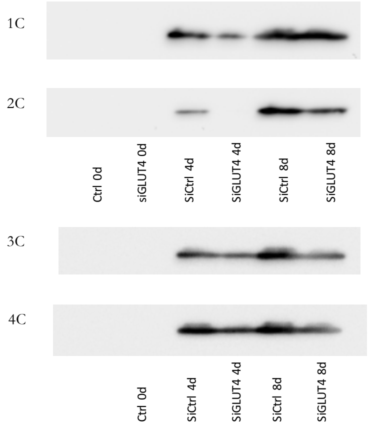

1C Merged FABP4

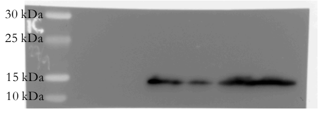

1.C, 2.C, 3C, 4C FABP4 #3544 1:1000 rabbit
NuPage 4-12%, 1mm, MOPS, BioRad transfer 30 min

Full length membarnes to Figure 3C
Perilipin protein

Some of the original membranes have been cut to enable blotting of multiple proteins at different molecular size.
The original tiff-files from caputre is full length.

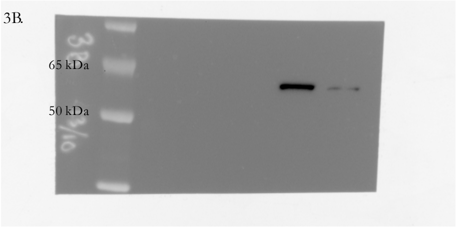

All quantified blotts

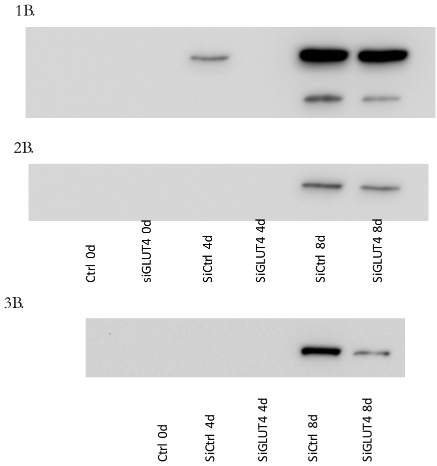

1.B och 2.B Perilipin #9349s 1:1000 rabbit
NuPage 4-12%, 1mm, MOPS, BioRad transfer 30 min
